# Supplementary material for: Hospital costs of Balloon Pulmonary Angioplasty (BPA) procedure and management for CTEPH patients: An observational study based on the French national hospital discharge database (PMSI)
Source: PLoS One. 2021 Dec 7;16(12):e0260483. doi: 10.1371/journal.pone.0260483 (PMC8651124; doi:10.1371/journal.pone.0260483)
Supplement: S1 File — (DOCX) [file pone.0260483.s001.docx]

**S1 File: ICD-10 codes of comorbidities**

| **ICD-10 codes** | **Wording (in French)** | **Comorbidities** | **Condition** |
| --- | --- | --- | --- |
| D57 | Affections à hématies falciformes [drépanocytaires] | Sickle-cell |  |
| D68 | Autres anomalies de la coagulation | Coagulation disorder | Except D683 |
| E03 | Autres hypothyroïdies | Hypothyroidism |  |
| E10 | Diabète sucré de type 1 | Diabete |  |
| E11 | Diabète sucré de type 2 | Diabete |  |
| E13 | Autres diabètes sucrés précisés | Diabete |  |
| E66 | Obésité | Obesity |  |
| I25 | Cardiopathie ischémique chronique | Chronic ischaemic heart disease |  |
| I50 | Insuffisance cardiaque | Heart failure |  |
| I51 | Complications de cardiopathies et maladies cardiaques mal définies | Heart failure | Only if associated with I50 during the same stay |
| J42 | Bronchite chronique, sans précision | Chronic respiratory disease |  |
| J43 | Emphysème | Chronic respiratory disease |  |
| J44 | Autres maladies pulmonaires obstructives chroniques | Chronic respiratory disease |  |
| J81 | Oedème pulmonaire | Chronic ischaemic heart disease | If recent diagnosis and associated with a cardiac pathology ( I20-I25 ; I30-I52) |
| J84 | Autres affections pulmonaires interstitielles | Chronic respiratory disease |  |
| J961 | Insuffisance respiratoire, non classée ailleurs | Chronic respiratory disease |  |
| K70 | Maladie alcoolique du foie | Liver disease |  |
| K76 | Autres maladies du foie | Liver disease |  |
| M32 | Lupus érythémateux disséminé | Connectivite |  |
| M35 | Autres atteintes systémiques du tissu conjonctif | Connectivite |  |
| N18 | Maladie rénale chronique | Chronic renal disease |  |
| R18 | Ascite | Ascites |  |
| Z950 | Implants et de greffes cardiaques et vasculaires | Pacemaker |  |
