# Supplementary material for: Hospital costs of Balloon Pulmonary Angioplasty (BPA) procedure and management for CTEPH patients: An observational study based on the French national hospital discharge database (PMSI)
Source: PLoS One. 2021 Dec 7;16(12):e0260483. doi: 10.1371/journal.pone.0260483 (PMC8651124; doi:10.1371/journal.pone.0260483)
Supplement: S2 File — (DOCX) [file pone.0260483.s002.docx]

S2 File: Medical transports method

***Ambulance tariffs***

The tariff of medical transports by ambulance decomposes as follows:

1. A department flat rate or an agglomeration flat rate or a management flat rate, depending on the location of the headquarters of the medical transport company
2. A short-distance valuation: degressive incresa, depending on the number of kilometers traveled (from 0 to 19 km)
3. A kilometric tariff: applicable to the distance traveled from the place of departure to the place of arrival, after deducting the first 3 km included (without deduction if the management flat rate applies)

Table 5: Tariffs of medical transports by ambulance (tariffs in euros applicable on 1 February 2013)

| Departmental flat rate | 51.30 € |
| --- | --- |
| Agglomeration flat rate | 57.37 € |
| Management | 64.30 € |
| Kilometric tariff | 2.19 € |
| Short-distance valuation ≤ 5 km traveled | 7.00 € |
| Short-distance valuation > 5 and ≤ 10 km traveled | 5.50 € |
| Short-distance valuation > 10 and ≤ 15 km traveled | 4.00 € |
| Short-distance valuation >15 and ≤ 19 km traveled | 2.50 € |

To take account of the municipalities, we valued as follows:

| 1. If one of the departments below 75, 92, 93, 94 or one of the municipalities in Annex 1^[[1]](#footnote-1)^ belongs to the processing hospital, then the valuation is carried out as follows:  ***Mean cost ambulance = 64.30 + short-distance valuation (if ≤ 19 km) + X*2.19/km***  2. If the transport is carried out within designed urban agglomerations in each department:  ***Mean cost ambulance = 57.37 + short-distance valuation (if ≤ 19 km) + (X-3)*2.19/km***  3. If another department (excluding FA and FPC) is chosen:  ***Mean cost ambulance = 51.30 + short-distance valuation (if ≤ 19 km) + (X-3)*2.19/km***  where X = mean distance “Hospital-Patient” |
| --- |

***LMV tariffs (Light Medical Vehicle)***

As the ambulances, LMS tariffs is therefore the following:

1. A department flat rate or an agglomeration flat rate or a management flat rate, depending on the location of the headquarters of the medical transport company
2. A short-distance valuation: degressive incresa, depending on the number of kilometers traveled (from 0 to 18 km)
3. A kilometric tariff: applicable to the distance traveled from the place of departure to the place of arrival, after deducting the first 3 km included (without deduction if the management flat rate applies)

Table 6: Tariffs of medical transports by LMV (tariffs in euros applicable on 1 January 2015)

| Departmental flat rate zone A | 13.85 € |
| --- | --- |
| Departmental flat rate zone B | 13.45 € |
| Departmental flat rate zone C | 12.60 € |
| Departmental flat rate zone D | 11.97 € |
| Management | 15.58 € |
| Kilometric tariff | 0.89 € |
| Short-distance valuation ≤ 7 km traveled | 6.26 € |
| Short-distance valuation > 7 and ≤ 8 km traveled | 6.05 € |
| Short-distance valuation > 8 and ≤ 9 km traveled | 5.53 € |
| Short-distance valuation > 9 and ≤ 10 km traveled | 5.00 € |
| Short-distance valuation > 10 and ≤ 11 km traveled | 4.48 € |
| Short-distance valuation > 11 and ≤ 12 km traveled | 3.96 € |
| Short-distance valuation > 12 and ≤ 13 km traveled | 3.44 € |
| Short-distance valuation > 13 and ≤ 14 km traveled | 2.92 € |
| Short-distance valuation > 14 and ≤ 15 km traveled | 2.40 € |
| Short-distance valuation > 15 and ≤ 16 km traveled | 1.88 € |
| Short-distance valuation > 16 and ≤ 17 km traveled | 1.36 € |
| Short-distance valuation > 17 and ≤ 18 km traveled | 0.83 € |

As the ambulances, the “management flat rate”, applies only to Paris and for certain agglomerations in the Paris region.

The “department flat rate” is intented for transports carried out in all municipalities other than those covered by the application of “management flat rate”.

The tariff applicable to each compagny is the department tariff where the headquarter of the company is situated; when the company carries out transports in other department, the tariff applicable remains that of the depatment of the headquarters of the company.

| 1. If one of the departments below 75, 92, 93, 94 or one of the municipalities in Annex 1^[[2]](#footnote-2)^ belongs to the processing hospital, then the valuation is carried out as follows:  ***Mean cost LMV = 15.58 + short-distance valuation (if ≤ 18 km) + X*0.89/km***  2. If the transport is carried out within the department 78, 91, 95 (Zone A), then:  ***Mean cost LMV = 13.85 + short-distance valuation (if ≤ 18 km) + (X-3)*0.89/km***  3. If the transport is carried out within the department 04, 06, 09, 67, 13, 20, 33, 31, 05, 74, 65, 38, 42, 44, 59, 62, 66, 69, 73, 77, 76, 83 (Zone B), then:  ***Mean cost LMV = 13.45 + short-distance valuation (if ≤ 18 km) + (X-3)*0.89/km***  4. If the transport is carried out within the department 01, 07, 10, 12, 14, 15, 16, 17, 19, 21, 23, 25, 26, 29, 30, 43, 87, 68, 34, 35, 37, 39, 45, 48, 49, 51, 54, 56, 57, 63, 64, 72, 80, 84 (Zone C), then:  ***Mean cost LMV = 12.60 + short-distance valuation (if ≤ 18 km) + (X-3)*0.89/km***  5. If the transport is carried out within the department 02, 03, 08, 11, 18, 22, 79, 24, 27, 28, 32, 52, 70, 36, 40, 41, 46, 47, 50, 53, 55, 58, 60, 61, 71, 81, 82, 90, 85, 86, 88, 89 (Zone D), then:  ***Mean cost LMV = 11.97 + short-distance valuation (if ≤ 18 km) + (X-3)*0.89/km***  where X = mean distance “Hospital-Patient” |
| --- |

***Taxi^[[3]](#footnote-3)^***

Medical transports by taxi must be carried out by licensed taxis.

The cost of a taxi fare is determined by the horokilometric meter (taximeter), which is mandatory for each taxi. The principe of operation of the taximeter is identical to all taxis, but the application of kilometric and hourly terms differs for the Parisian or provincial taxis.

The cost of a taxi fare is divided into 3 elements:

1. Management (Day/Night); i.e. the starting cost of the meter when it is switched on
2. KM: tariff for one kilometer
3. TIME: hourly tariff automatically engages when the meter is running and the vehicle is stopped (traffic jam, customer waiting) or when the kilometer tariff is equivalent with the hourly tariff (speed of the vehicle less than the hourly tariff divided by the kilometric tariff applied)

Difference exist between Parisian taxis and provincial taxis (Appendix 2).

*Paris taxis*

1. KM A and TIME A
   - Applicable in Paris intramural peripheral boulevard included from 10am to 5pm
2. KM B and TIME B
   - Applicable in Paris intramural peripheral boulevard included from 5pm to 10am
   - Applicable in Paris intramural peripheral boulevard included from 7am to 24pm on Sundays
   - Applicable in Paris intramural peripheral boulevard included from 0am to 7am on public holidays
   - Applicable every day in suburban zone (the suburban zone is composited of the municipalities outside Paris, but attached to Parisian taxis) from 7am to 7pm
3. KM C and TIME C
   - Applicable in Paris intramural peripheral boulevard included from 0am to 7am on Sundays
   - Applicable in the suburban zone from 7pm to 7am
   - Applicable in the suburban zone at all times on Sundays and public holidays
   - Applicable every day and any time beyond the suburban zone

*Taxis, other areas*

1. KM A
   - Applicable for a round trip during the day from Monday to Saturday included
2. KM B
   - Applicable for a round trip during the night from Monday to Saturday included or day and night on Sundays and public holidays
3. KM C
   - Applicable for a one-way trip during the day from Monday to Saturday included
4. KM D
   - Applicable for a one-way trip during the night from Monday to Saturday included or day and night on Sundays and public holidays

Day or night schedules vary from one department to another, but are most often the followings:

- - Day: 7am – 7pm or 8am – 8pm
  - Night: 7am – 7pm or 8am – 8pm

Whatever the amount displayed on the meter, the sum to be paid cannot be less than 7€.

Approximations were carried out for the valuation of this transport mode. We chose to value the fare, according to the kilometric distance, and this in the absence of information on the weight of the hourly cost of the taxi, and we made the assumption that this fare corresponded to a fare carried out the week, the day.

With these assumptions, the calculation carried out as follows:

***Mean cost taxi = Management + distance traveled * KM C (A if Paris)***

***If the sum is less than 7€, then the latter tariff is due to the insured.***

Note:

NHI only reimburses taxis. It is quite possible that it has entered into an agreement with specific tariffs. It should be also noted that if the taxi fare is greater than a management by LMV, then the patient is reimbursed on the basis of the LMV cost.

***Public transport tariffs***

We uses the repositories proposed by UBS^[[4]](#footnote-4)^, which allows to obtain a large number of indicators on the service costs, especially public transports, in the main cities wordwide. For France, only 2 cities, Lyon and Paris have a tariff reference for a trip of 10 km.

| Lyon | 1.82 € |
| --- | --- |
| Paris | 1.82 € |
| Mean | 1.82 € |

The mean of 1.82€ for 10 km by public transport was used.

***Individual transport tariffs***

Other individual transport modes can be reimbursed:

1. Passenger cars
2. Mopeds and motorcycles

For patients using this transport mode, we assumed that these patients have received a reimbursement for a passenger car.

Since March 30^th^, 2015^[[5]](#footnote-5)^, the kilometer tariff used on the basis for reimbursement by the NHI is set at 0.30€.

1. List of municipalities concerned:

   <http://www.ameli.fr/fileadmin/user_upload/documents/Liste_des_communes_visees_par_le_forfait_agglomeration__format_PDF_.pdf>, cited 01/FEB/2017 [↑](#footnote-ref-1)
2. <http://www.ameli.fr/professionnels-de-sante/transporteurs/votre-convention/tarifs/vsl-les-tarifs-conventionnels/communes-concernees-par-la-prise-encharge.php>, cited 01/FEB/2017 [↑](#footnote-ref-2)
3. <http://www.taxis-de-france.com/professionnel/tarifstaxis.htm>, cited 01/FEB/2017 [↑](#footnote-ref-3)
4. <https://itunes.apple.com/fr/app/ubs-prix-salaires/id453181204?mt=8>, cited 01/FEV/2017 [↑](#footnote-ref-4)
5. Decret of March 30, 2015 fixing the tariff used as the basis for the reimbursement by the National Health Insurance of individual modes refered to II of Article R. 322-10-5 of Social Security code. [↑](#footnote-ref-5)
