# Supplementary material for: Hospital costs of Balloon Pulmonary Angioplasty (BPA) procedure and management for CTEPH patients: An observational study based on the French national hospital discharge database (PMSI)
Source: PLoS One. 2021 Dec 7;16(12):e0260483. doi: 10.1371/journal.pone.0260483 (PMC8651124; doi:10.1371/journal.pone.0260483)
Supplement: Attachment — Submitted filename: 2021-10-21_BPA_PMSI_Response to Reviewers.docx [file pone.0260483.s004.docx]

**Comments to the Author**

Please use the space provided to explain your answers to the questions above. You may also include additional comments for the author, including concerns about dual publication, research ethics, or publication ethics. (Please upload your review as an attachment if it exceeds 20,000 characters)

**Reviewer #1: The authors have adequately addressed my concerns.**
This paper will be an important contribution to the field.

**Reviewer #2:**

This is an interesting paper that deserves to be published. However,

1. I do not agree with the response to question 2. One cannot negate that drugs are part of CTEPH management. Therefore, drugs should be mentioned in the introduction, including their availability in France. Authors will need to clarify that drugs costs are not subject of the present analysis as they were paid out-of-hospital.

**Response to answer 1  to reviewer 2:**

We added the drug authorized for CTEPH management in the introduction part (page 3, lines 64-66) as above :

*“Riociguat, an oral guanylate cyclase stimulator, and treprostinil, a subcutaneous prostacyclin analogue, are approved (in 2014 and 2020 respectively) for patients with inoperable CTEPH or persistent/recurrent PH after PEA; other PH medications are also off-label used(6)”*

We also precised in the methodology part of the manuscript that out of hospital drugs are not analysed in the present study (page 7, lines 158-159):

*“Out of hospital drugs were not included in the present analysis as they are not recorded in the database.”*

6. Delcroix M, Torbicki A, Gopalan D, Sitbon O, Klok FA, Lang I, et al. ERS statement on chronic thromboembolic pulmonary hypertension. Eur Respir J. 2021 Jun;57(6):2002828.

2. I realized that the important message within authors‘ responses that …intensive care unit are the major drivers of hospital cost in the present study….cannot easily be found in the manuscript. Please, add those cost for intensive care stays to Table 2.

**Response to answer 2 to reviewer 2:**

We agree that this information is interesting to add. Therefore, we added the following relevant results regarding ICU in table 2 : number of stays with ICU transit ; mean length of stay in ICU, part of ICU cost among total cost, mean ICU cost/stay and mean ICU cost/patient. We also modified the text in consequence to describe these results.
